# Easy ensemble classifier-group and intersectional fairness and threshold (EEC-GIFT): a fairness-aware machine learning framework for lung cancer screening eligibility using real-world data

**DOI:** 10.1093/jncics/pkaf030

**Published:** 2025-03-20

**Authors:** Piyawan Conahan, Lary A Robinson, Trung Le, Gilmer Valdes, Matthew B Schabath, Margaret M Byrne, Lee Green, Issam El Naqa, Yi Luo

**Affiliations:** Department of Machine Learning, H. Lee Moffitt Cancer Center and Research Institute, Tampa, FL, United States; Division of Thoracic Oncology (Surgery), H. Lee Moffitt Cancer Center and Research Institute, Tampa, FL, United States; Department of Industrial and Management Systems Engineering, University of South Florida, Tampa, FL, United States; Department of Machine Learning, H. Lee Moffitt Cancer Center and Research Institute, Tampa, FL, United States; Division of Thoracic Oncology (Surgery), H. Lee Moffitt Cancer Center and Research Institute, Tampa, FL, United States; Department of Cancer Epidemiology, H. Lee Moffitt Cancer Center and Research Institute, Tampa, FL, United States; Department of Health Outcomes and Behavior, H. Lee Moffitt Cancer Center and Research Institute, Tampa, FL, United States; Department of Health Outcomes and Behavior, H. Lee Moffitt Cancer Center and Research Institute, Tampa, FL, United States; Department of Machine Learning, H. Lee Moffitt Cancer Center and Research Institute, Tampa, FL, United States; Department of Machine Learning, H. Lee Moffitt Cancer Center and Research Institute, Tampa, FL, United States

## Abstract

**Background:**

We use real-world data to develop a lung cancer screening (LCS) eligibility mechanism that is both accurate and free from racial bias.

**Methods:**

Our data came from the Prostate, Lung, Colorectal, and Ovarian (PLCO) cancer screening trial. We built a systematic fairness-aware machine learning framework by integrating a Group and Intersectional Fairness and Threshold (GIFT) strategy with an easy ensemble classifier—(EEC-) or logistic regression—(LR-) based model. The best LCS eligibility mechanism EEC-GIFT* and LR-GIFT* were applied to the testing dataset and their performances were compared to the 2021 US Preventive Services Task Force (USPSTF) criteria and PLCO_M2012_ model. The equal opportunity difference (EOD) of developing lung cancer between Black and White smokers was used to evaluate mechanism fairness.

**Results:**

The fairness of LR-GIFT* or EEC-GIFT* during training was notably greater than that of the LR or EEC models without greatly reducing their accuracy. During testing, the EEC-GIFT* (85.16% vs 78.08%, *P *<* *.001) and LR-GIFT* (85.98% vs 78.08%, *P *<* *.001) models significantly improved sensitivity without sacrificing specificity compared to the 2021 USPSTF criteria. The EEC-GIFT* (0.785 vs 0.788, *P *=* *.28) and LR-GIFT* (0.785 vs 0.788, *P *=* *.30) showed similar area under receiver operating characteristic curve values compared to the PLCO_M2012_ model. While the average EODs between Blacks and Whites were significant for the 2021 USPSTF criteria (0.0673, *P *<* *.001), PLCO_M2012_ (0.0566, *P *<* *.001), and LR-GIFT* (0.0081, *P *<* *.001), the EEC-GIFT* model was unbiased (0.0034, *P *=* *.07).

**Conclusion:**

Our EEC-GIFT* LCS eligibility mechanism can significantly mitigate racial biases in eligibility determination without compromising its predictive performance.

## Introduction

Lung cancer is the leading cause of cancer-related death in the United States. In 2024, lung cancer accounted for an estimated 20% of all cancer-related deaths.^1^ The National Lung Screening Trial demonstrated that lung cancer screening (LCS) with low-dose computed tomography (LDCT) can detect early-stage lung cancers, reducing lung cancer mortality by up to 20%.^2^ In the first step of the LCS process, a screening eligibility model identifies individuals most likely to benefit from the screening.

Initially, the 2013 US Preventive Services Task Force (USPSTF) criteria were used to determine eligibility for LCS, which included age (55-80 years) and smoking history (≥30 pack-year, current smoker, or quit within the past 15 years).^3^ Although these criteria cover many eligible White smokers, they miss many high-risk Black smokers,^4^ who often develop lung cancer at younger ages, with less smoking intensity, and at more advanced stages.^5-8^ In 2021, USPSTF updated its guidelines by lowering the age threshold from 55 to 50 and reducing the pack-year from 30 to 20.^9^ While these changes have reduced racial disparities, the age and smoking history-based criteria lead to limited incidence predictive performance. As such, there still may be a risk of getting a high false positive rate and false negative rate (FNR) for Black and White individuals based on the 2021 USPSTF criteria.^10^^,^^11^

Recent studies aim to develop more accurate LCS eligibility policies. Developing risk-based eligibility models that estimate individuals’ lung cancer risk and set screening thresholds is promising—especially since machine learning (ML) can revolutionize personalized healthcare and improve early cancer detection using multidimensional datasets.^8^ Many models, such as the PLCO_M2012_ model^12^ and its variants, used logistic regression (LR) to analyze relationships among smokers’ demographic information, smoking history, personal history of any cancer, family history of lung cancer, lung-related diseases, and their lung cancer incidence from retrospective datasets. Results have shown that, compared with the 2021 USPSTF criteria, incorporating additional information can improve lung cancer risk prediction with more sensitivity or specificity within certain racial/ethnic groups.^8^^,^^12^ However, the characteristics used to predict lung cancer in these models may not be independent, and their relationship with the disease may be non-linear in clinical practice. Also, incidence predictive models which are trained on datasets with inherent racial biases can perpetuate these biases, leading to racial disparities in lung cancer predictive performance. Moreover, imbalanced Black and White classes in the dataset can provide additional racial disparities in the incidence predictive accuracy, which may further increase the level of over-diagnosis or under-diagnosis of lung cancer within certain racial/ethnic groups.

Algorithmic fairness refers to the un-coverage and rectification of biases along social axes such as race, gender, and class in automated decision processes from ML-based predictive models.^13-15^ LCS eligibility decisions made by such models after a learning process may be considered unfair if these decisions were based on sensitive attributes (eg, race, gender). When a healthcare organization chooses an appropriate ML–based lung cancer prediction and racial bias mitigation model for clinical decision-making regarding LCS eligibility, both the accuracy of lung cancer prediction and its fairness should be considered simultaneously. Though risk-based eligibility models (eg, the PLCO_M2012_ model) are promising for determining LCS eligibility, their fairness across different racial groups, particularly between Black and White smokers, remains underexamined.^16^ This gap poses a significant barrier to effectively integrating these models into clinical practice. In response, we sought to develop a systematic fairness-aware ML framework for an accurate and racially unbiased LCS eligibility model.

An important preliminary step for our research was to determine the best strategy for defining and evaluating fairness. Group fairness is a popular approach for treating 2 groups equally. With this approach, groups are typically identified by sensitive attributes, such as race or gender, and quantities are compared at the group level. Group fairness criteria mainly include demographic parity, equalized odds, and equal opportunity.^17^ A major shortcoming of group fairness is that it does not prevent unfairness against those who are found at the intersection of multiple types of discrimination (eg, Black females). Conversely, intersectional fairness explores the biases that might be encoded in ML models by considering different subgroups that overlap and intersect across multiple dimensions,^18^ potentially improving fairness and preventing fairness gerrymandering, which is the substantial violation of the fairness constraint on 1 or more structured subgroups defined over the protected attributes (eg, Black females).^19^

Additionally, it was crucial to identify our core bias mitigation approaches for fairness. Strategies for bias mitigation can be broadly classified as pre-processing, in-processing, or post-processing,^20^ each of which targets different stages of the ML model development pipeline to ensure the fairness and equity of its outcomes. Pre-processing methods tackle bias by adjusting the training data before model development^21^; in-processing methods modify the learning algorithms during the training process^22^; and post-processing methods adjust the model’s outputs after training to achieve fairer outcomes.^23^

Building on these insights, we developed a Group and Intersectional Fairness and Threshold (GIFT) fairness-enhancing strategy by integrating both pre- and post-processing bias mitigation approaches. To achieve a racially unbiased LCS eligibility mechanism with intersectional fairness, we defined multiple types/combinations of discrimination, which always included race. We applied the reweighing method for group fairness to intersectional fairness as a pre-processing approach,^21^ and then determined a risk threshold from different risk decision boundaries as a post-processing method.^23^

However, though this integration was devised to ensure racial parity, any bias mitigation approach will inevitably affect the accuracy of ML models.^24^ The GIFT fairness-enhancing strategy is not an exception, especially in handling the National Cancer Institute Prostate, Lung, Colorectal, and Ovarian (PLCO) cancer screening trial datasets^25^ with imbalances in both the number of participants with and without lung cancer and the distribution of Black and White participants. Given that these existing imbalances cannot be changed, it is crucial to choose an appropriate ML model to mitigate biases due to the imbalances and achieve a high lung cancer predictive performance. Typically, decision tree–based algorithms, such as bagging- and boosting-based techniques, perform well on imbalanced datasets.^26^ Resampling the imbalanced data before entering it into an ML model can help better capture the decision boundary of lung cancer incidence (Yes/No). Therefore, an easy ensemble classifier (EEC) model,^27^ which combines random under-sampling and AdaBoost classifier, is a suitable approach to handle imbalanced datasets and improve the accuracy of lung cancer prediction. In the EEC model, random under-sampling is designed to improve the accuracy for less frequently represented groups without significantly affecting predictions for the majority group. Therefore, we developed a systematic fairness-aware ML framework, named EEC-GIFT, to identify a new LCS eligibility mechanism from real-world data by embedding the GIFT strategy into the EEC-based lung cancer prediction model. All technical terms used throughout this paper are explained by a glossary in Table S1.

## Methods

### Assumptions and algorithmic fairness measurement

Because our study used real-world data, our fairness analysis was conducted with two major assumptions. Statistical parity difference (SPD) was used to evaluate the difference of the probabilities of lung cancer diagnosis (positive outcomes) between Black and White groups with a value of 0 indicating fairness. However, if the base rates of White and Black participants were significantly different, a fully accurate classifier to satisfy SPD = 0 may be considered unfair. Thus, our first assumption was that *the base rates of Black and White smokers developing lung cancer were not significantly different*. Furthermore, we used equal opportunity to define fairness across Black and White groups based on their true positive rates (TPRs)/FNRs and average equal opportunity difference (EOD) to measure and compare the fairness of LCS eligibility mechanisms (Supplementary Methods). If the base rates of Black and White participants developing lung cancer are not representative of the “true” values and were obtained with bias, a fully accurate classifier to satisfy this equal opportunity constraint may be considered unfair. Therefore, our second assumption was that *Black and White participants are representative of the true real-world population, and their data were not obtained in a biased manner*.

### Training and testing of the EEC-GIFT–based LCS eligibility model

**Figure 1.**
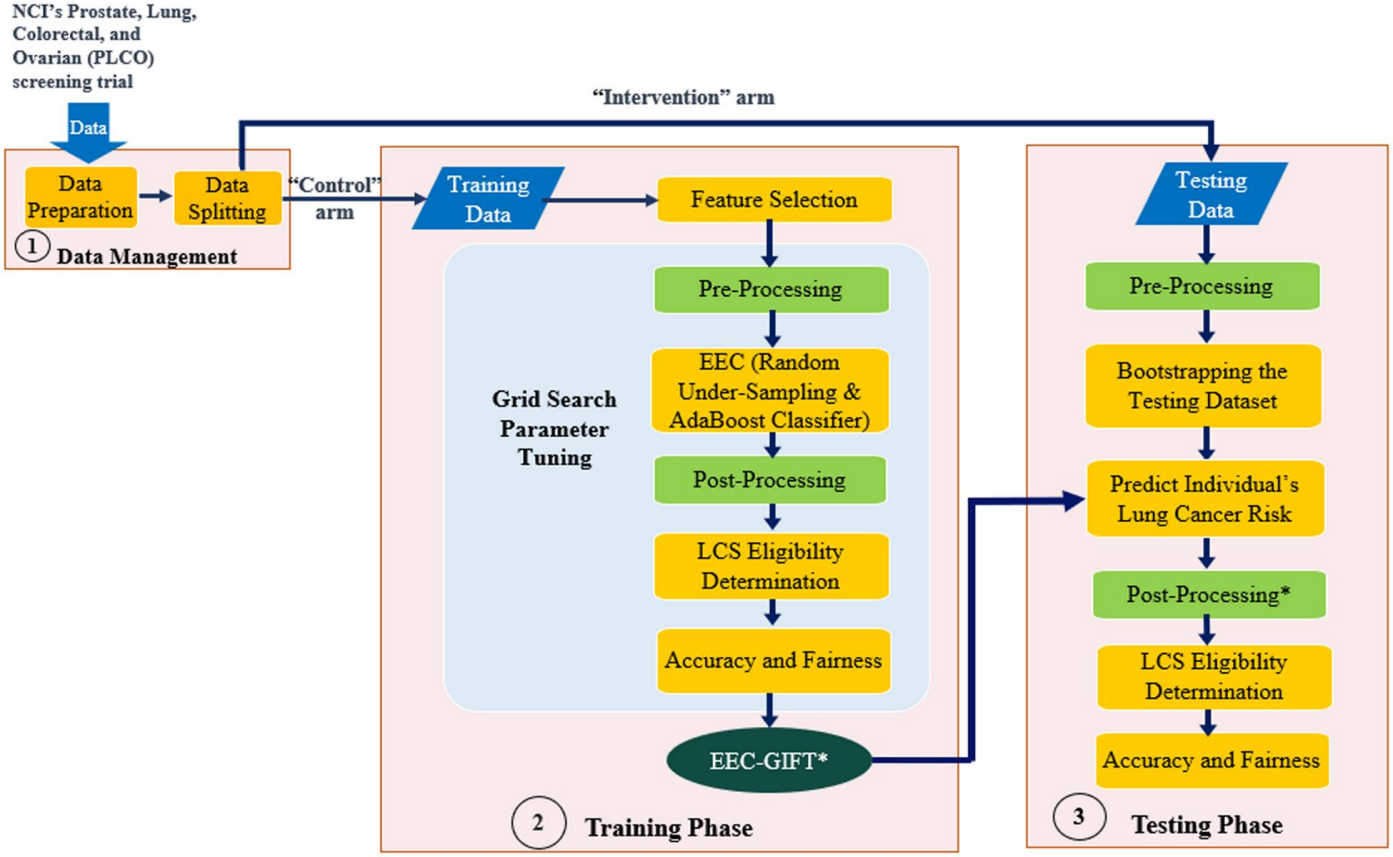
Training and testing an EEC-GIFT–based LCS eligibility mechanism. The best EEC-GIFT*–based LCS eligibility model can be trained by performing a grid search from stratified 10-fold cross-validation (CV) based on the training dataset to identify the optimal pre-processing strategy, the best parameters of the EEC in the EEC-GIFT*-based LCS eligibility model, and the best post-processing threshold in terms of both accuracy and fairness. Then, we applied the EEC-GIFT* model to our testing dataset to evaluate its accuracy and fairness. The details of the “Pre-Processing” reweighing strategy (training and testing) and “easy ensemble classifier (EEC)” (training) are introduced in Supplementary Methods. The “Post-Processing” threshold strategy (training) is introduced in Figure S1. The evaluations and statistical tests of “Accuracy and Fairness” (training and testing) are introduced in the Statistical Analysis section.

Our study was conducted based on the PLCO trial, where institutional review board approvals were received at all 10 sites. Informed consent was secured, and participants completed self-reported questionnaires on demographics and medical history at baseline and throughout the study.^25^ After performing data preparation and feature selection (Supplementary Methods), the EEC-GIFT–based LCS eligibility model was developed by integrating pre- and post-processing bias mitigation strategies and embedding them into the EEC–based lung cancer prediction model (Supplementary Methods) as illustrated in Figure 1. The pre-processing reweighing approach allows multiple sensitive attributes, and the post-processing strategy permits numerous threshold options to determine eligibility. Let ${“T}_{U}”$ be the threshold of LCS eligibility associated with the specificity of the 2021 USPSTF criteria, and “$T_{Y}”$ be the threshold of LCS eligibility associated with the Youden Index^28^ of the ROC curve of the incidence prediction model (Supplementary Methods**)**. To proof-of-concept, we trained the best EEC-GIFT* LCS eligibility model by considering 1 (“race”) and 2 (“race” and “gender”) sensitive attributes for the pre-processing reweighing approach and assigning 2 thresholds (“$T_{U}$” and “$T_{Y}$”) for the post-processing strategy. Additionally, we trained the best LR_GIFT*–based prediction model based on the features in the PLCO_M2012_ model and the GIFT strategy with the same sensitive attributes and threshold strategy. Then we tested the EEC-GIFT* and LR-GIFT* models and compared their accuracy and fairness to other LCS eligibility mechanisms.

### Statistical analysis

We evaluated and compared the accuracy and fairness of the USPSTF criteria, PLCO_M2012_, LR-GIFT*– and EEC-GIFT*–based models. Since the USPSTF standard is a rule–based model with binary outcomes, we used sensitivity and specificity to evaluate its predictive performance and employed McNemar’s test^29^ to compare it with other LCS eligibility models. For risk–based models, we used area under the receiver operating characteristic curves (AUCs) to evaluate their accuracy and employed Delong’s test^30^ to compare their performance. All statistical tests were performed at a significance level of 0.05.

Fairness was assessed by comparing Black and White participants’ TPRs. Specifically, we used a paired t-test to evaluate a hypothesis of H_0_: average EOD = 0 and H_1_: average EOD ≠ 0. If *P *<* *.05, H_0_ was rejected and the Black and White groups’ TPRs were considered significantly different. Otherwise, H_0_ was failed to reject and LCS eligibility was considered fair. In addition, we used Hedges’ *g*_rm_ to calculate the size of this difference or effect sizes^31^ and employed the common language (CL) effect size^32^ to quantify the magnitude of disparities. Hedges’ *g*_rm_ offers a bias-corrected measure of the standardized difference, where values below 0.2 indicate a small effect, around 0.5 a moderate effect, and above 0.8 a large effect. The CL effect size translates this into the probability that a randomly selected individual from one group outperforms one from another group. Together, these metrics complement the t-test by highlighting both statistical significance and practical relevance.

## Results

### Data pre-preparation for the training and testing datasets

**Figure 2.**
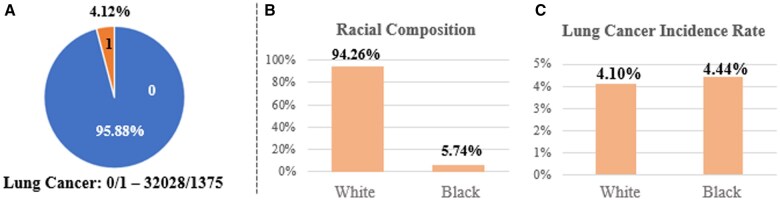
The properties of our training data. (A) Shows that the number of participants who were confirmed to have lung cancer was only 4.12% of the whole population in our training dataset. (B) Illustrates that Black participants comprise only 5.74% of all participants in the training dataset. These class imbalance issues can affect the accuracy and fairness of ML-based LCS eligibility models. (C) Shows racial disparities in lung cancer incidence rates between Black (4.44%) and White (4.10%) smokers in the training dataset. However, a 2-proportion z-test confirms these incidence rates are not significantly different (*P* = .47), which satisfies the assumption of our study.

The original PLCO lung dataset includes 154 887 participants and 251 variables. Given Black and White smokers have higher lung cancer incidence and mortality rates than any other races,^5^ we focused our analysis on these 2 subgroups. We further narrowed the PLCO dataset to only include current or previous Black and White smokers who had completed the baseline questionnaire, had no history of lung cancer prior to the PLCO screening trial, and did not have any missing incidence information. This resulted in 67 612 participants and 2844 confirmed lung cancer cases. To avoid possible effects of overdiagnosis bias^33^^,^^34^ and align with the training and testing processes of other risk-based LCS eligibility models in the literature,^12^^,^^35^ we used the PLCO control arm (33 403 participants) to train the models and its intervention arm (34 209 participants) to test the models. The properties of our training dataset are illustrated in Figure 2.

### Best LCS eligibility mechanisms in the training phase

The selected features used to develop the EEC– and EEC-GIFT–based LCS eligibility mechanisms are shown in Table 1. Let RW1 and RW2 represent reweighing approaches associated with 1 (“race”) and 2 (“race” and “gender”) sensitive attributes, respectively. The SPD values and group/subgroup weight coefficients based on pre-processing strategies RW1 (Table S2) and RW2 (Table S3) were computed by using the pre-processing reweighting formulas (Supplementary Methods**)**. The post-processing strategy of identifying thresholds “$T_{U}$” and “$T_{Y}$” based on an ROC curve is illustrated in Figure S1. The accuracy and fairness of different LCS eligibility mechanisms in the training phase are shown and compared in Table 2, where the best LCS eligibility models “EEC_RW1 & $T_{U}$” and “LR_RW1 & $T_{U}$” were subsequently tagged as “EEC-GIFT*” and “LR-GIFT*,” respectively. Important features to determine lung cancer risk based on the EEC-GIFT* model are shown in Figure S2.

**Table 1.** List of selected features to develop the EEC- and EEC-GIFT–based LCS eligibility mechanisms.

Categories	Features
Demographic Factors	(1) age, (2) education level, (3) BMI
Smoking History	(4) smoking status, (5) pack-years, (6) number of years smoked, (7) number of years since stopped smoking
Lung-Related Issues	(8) COPD (aggregation of “bronchitis” and “emphysema”),^a^ (9) Personal Health History (aggregation of “family history of lung cancer,” “personal history of any cancer,” and “chest X-ray history”)^a^

a The details of aggregated features are introduced in data preparation (Supplementary Methods).

**Table 2.** The comparisons of different LCS eligibility mechanisms in the training phase.^a^

Performance		LR	LR_RW1	LR_RW2	EEC	EEC_RW1	EEC_RW2
AUC & 95% CI (Whole)^b^		0.783 (0.772 to 0.795)	**0.783** **(0.772 to 0.794)**	0.783 (0.771 to 0.794)	0.793 (0.782 to 0.804)	**0.791** **(0.780 to 0.802)**	0.792 (0.781 to 0.803)
AUC & 95% CI (Black)^b^		0.747 (0.700 to 0.794)	0.747 (0.700 to 0.794)	0.747 (0.700 to 0.794)	0.755 (0.705 to 0.804)	0.752 (0.704 to 0.801)	0.753 (0.704 to 0.802)
AUC & 95% CI (White)^b^		0.785 (0.774 to 0.797)	0.785 (0.774 to 0.797)	0.785 (0.773 to 0.796)	0.795 (0.785 to 0.806)	0.793 (0.782 to 0.804)	0.794 (0.783 to 0.805)
Avg. EOD & 95% CI ^c^	$T_{U}$ ^d^	0.024 (−0.05 to 0.093)	−**0.014** **(**−**0.105 to 0.068)**	−0.019 (−0.099 to 0.0558)	−0.028 (−0.101 to 0.051)	−**0.014** **(**−**0.096 to 0.06)**	−0.021 (−0.099 to 0.045)
	$T_{Y}$	−0.043 (−0.14 to 0.049)	−0.060 (−0.161 to 0.032)	−0.056 (−0.16 to 0.041)	−0.033 (−0.124 to 0.061)	−0.030 (−0.12 to 0.064)	−0.036 (−0.122 to 0.0442)

a Bold value represents the best LR-GIFT*- and EEC-GIFT*-based LCS eligibility mechanisms at the training phase based on the lowest average EODs and no significantly reduced predictive performance. The tuned pre-processing strategy was RW1, and the tuned post-processing threshold was “$T_{U}$.” The best parameters of the EEC in the EEC-GIFT*-based LCS eligibility model consisted of 70 different AdaBoost learners, 200 weak learners for each AdaBoost ensemble, and learning rate of 0.1. Though the average EODs of LR-GIFT*- and EEC-GIFT*-based LCS eligibility mechanisms were the same, the 95% CI of the EEC-GIFT* was narrower than that of the LR-GIFT*, indicating that the EEC-GIFT* has better fairness.

b The predictive performance of different LCS eligibility mechanisms based on 10-fold stratified CV was evaluated using the AUCs with 95% CI via stratified bootstrap sampling. The 2nd row of the table shows the predictive performance of each LCS eligibility model. After separating the training dataset into Black and White subgroups, we applied the trained LCS eligibility models to each subgroup to validate their predictive performance. The 3rd and 4th rows show the performance of these eligibility models for black and white groups, respectively.

c The 5th and 6th rows illustrate the fairness of these eligibility models using thresholds “$T_{U}$” and “$T_{Y}$,” respectively. Although the LR-GIFT* or the EEC-GIFT* has similar prediction performance as the LR and “LR_RW2 & $T_{U}$” models or the EEC and “EEC_RW2 & $T_{U}$” models (*P  *>* *.05), their fairness was significantly higher (*P *<* *.001) based on independent *t*-tests. This indicates that the GIFT strategy can greatly improve the fairness of ML-based LCS eligibility models.

d We evaluated the values of “$T_{U}$” for the EEC-GIFT* or the LR-GIFT* from their ROC curves during the training phase by using the methods introduced in Figure S1. At the testing phase, we applied the EEC-GIFT* and the LR-GIFT* models with these thresholds to the testing dataset and compare their accuracy and fairness with the 2021 USPSTF criteria and the PLCO_M2012_ based LCS eligibility model.

### Comparison of different LCS eligibility mechanisms during the testing phases

**Figure 3.**
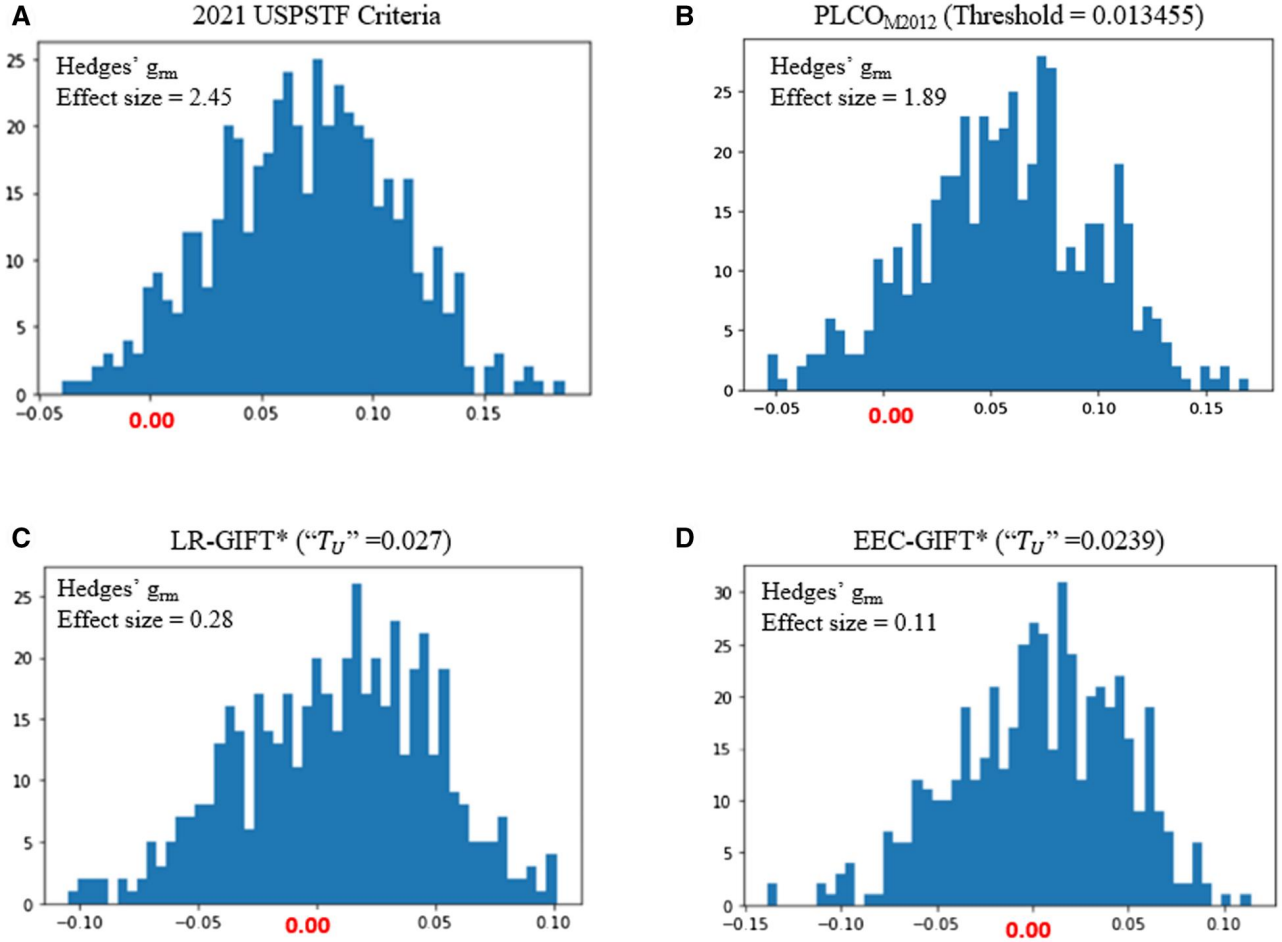
The EOD distributions of (A) 2021 USPSTF criteria, (B) PLCO_M2012_ model (threshold = 0.013455), (C) LR-GIFT* (“$T_{U}$” = 0.027) (D) EEC-GIFT* (“$T_{U}$” = 0.0239)—based LCS eligibility mechanisms. The comparison of the EOD distributions of the LR-GIFT* and EEC-GIFT* to the 2021 USPSTF criteria and PLCO_M2012_ model shows that the GIFT strategy can greatly improve the fairness of ML-based incidence predictive models. With the same GIFT strategy, the fairness of the EEC-GIFT* is better than that of the LR-GIFT*, since the class imbalance issue had been solved during the process of developing the EEC model. Due to biases in the PLCO data collection, White smokers are an unfair group with a lower average TPR compared to the Black group. Our numerical experiments show that, for lung cancer diagnosis in the unfair group, the EEC-GIFT* LCS eligibility model can lead to about 10% reduction of the FNR (early detection of 104 more smokers with lung cancer) compared to the 2021 USPSTF criteria, and 5% reduction of the FNR (early detection of 46 more smokers with lung cancer) compared to the PLCO_M2012_ model based on the testing dataset.

We bootstrapped 80% of the testing dataset 500 times and used this dataset to test and compare the accuracy and fairness of the trained LR-GIFT*– and EEC-GIFT*–based LCS eligibility mechanisms with that of the 2021 USPSTF criteria and the PLCO_M2012_–based eligibility model. Figure 3 illustrates the distributions of EODs between Black and White participants for the different LCS eligibility mechanisms. Table 3 shows that only the EEC-GIFT* model could significantly improve the fairness of LCS eligibility without compromising the accuracy of lung cancer prediction.

**Table 3.** The performance of 4 different LCS eligibility mechanisms during the testing phase.

Performance	Measurement	2021 USPSTF Criteria	PLCO_M2012_ (Threshold= 0.013455)^b^	LR-GIFT* (“$T_{U}$” =0.0270)	EEC-GIFT* (“ $T_{U}$ **” =0.0239)**
Accuracy^c^	Sensitivity	78.08%	NA^a^	85.98%	85.16%
	Specificity	56.48%	NA^a^	56.01%	56.70%
	F1-Score	13.60%	NA^a^	14.74%	14.80%
	AUC and 95% CI (Whole)	NA^a^	0.788 (0.777 to 0.797)	0.785 (0.775 to 0.796)	**0.785** **(0.774 to 0.796)**
	AUC and 95% CI (Black)	NA^a^	0.770 (0.719 to 0.819)	0.777 (0.728 to 0.818)	**0.775** **(0.723 to 0.820)**
	AUC and 95% CI (White)	NA^a^	0.789 (0.779 to 0.800)	0.786 (0.776 to 0.797)	**0.786** **(0.774 to 0.797)**
Fairness^d^	Average EOD and 95% CI	0.0673 (0.002 to 0.134)	0.0566 (−0.015 to 0.122)	0.0081 (−0.059 to 0.0718)	**0.0034** **(**−**0.07 to 0.067)**
$H_{0}:\mathrm{EOD}=0$ $H_{1}:\mathrm{EOD}\neq 0$	Paired *t*-test	*P *<* *.001 Reject H_0_	*P *<* *.001 Reject H_0_	*P *<* *.001 Reject H_0_	*P* **=**** **.07** **Fail to reject H_0_**
	Effect Size (Hedges’ *g*_rm_)	2.45	1.89	0.28	**0.11**
	CL Effect Size	0.95	0.92	0.58	**0.53**

a At the testing phase, we would like to evaluate the predictive performance of the risk-based LR-GIFT* and EEC-GIFT* LCS eligibility models, and compare them to that of the 2021 USPSTF criteria and the PLCO_M2012_ model. While the PLCO_M2012_ is also a risk-based model and the AUC can be applied for its predictive performance evaluation, the AUC measurement cannot be applied to the 2021 USPSTF criteria given it is a rule-based LCS eligibility model with binary outcomes (yes or no). Therefore, we only compared our new LCS eligibility models to the PLCO_M2012_ based on the AUCs and to the USPSTF criteria based on the sensitivity and specificity analysis.

b “Threshold= 0.013455” is the threshold associated with the PLCO_M2012_ model from literature,^12^ and it was used in our study to evaluate the TPRs for its fairness measurement.

c After ensuring the specificity of LR-GIFT* and EEC-GIFT* models were similar to that of the USPSTF criteria, we calculated and compared their sensitivities to those of the USPSTF standard using McNemar’s test at a significance level of 0.05. Compared to the 2021 USPSTF criteria, both the LR-GIFT* (*P *<* *.001) and EEC-GIFT* (*P *<* *.001) models demonstrated significantly enhanced sensitivity and similar specificity for lung cancer prediction. We also evaluated and compared the accuracy of the LR-GIFT* and EEC-GIFT* models to that of the well-known standard PLCO_M2012_ model in terms of whole AUC and its 95% CIs (2nd-5th rows) and found similar predictive performance. Additionally, we separated the testing dataset into Black and White subgroups and applied the PLCO_M2012_, LR-GIFT*, and EEC-GIFT* models to each subgroup to validate their prediction performance (AUCs), and we used stratified bootstrap sampling to compute their associated 95% CIs (6th-7th rows). It turns out that racial disparities in lung cancer prediction accuracy between Black and White smokers decreased with the GIFT bias mitigation strategy.

d After calculating the average EOD and its 95% CI for each model across 500 bootstrapped testing datasets, we determined that the EODs of the LCS eligibility models were normally distributed based on the Kolmogorov-Smirnov test and used paired *t*-tests to evaluate whether Black and White participants’ opportunity to qualify for LCS (or their average TPRs) were equal for each LCS eligibility model (9th-10th rows**)**. The average EODs between Black and White participants (8th row) were significant for the 2021 USPSTF criteria, PLCO_M2012_ model (threshold = 0.013455), and LR-GIFT*–based LCS eligibility model (*P *<* *.05); however, only the EEC-GIFT* model achieved equal opportunity (average EOD = 0.0034) between these two racial groups (*P *>* *.05). Additionally, the 9th and 10th rows show that EEC-GIFT* model yielded the smallest effect size of the EOD, with Hedges’ *g*_rm_ = 0.11 and the CL effect size = 0.53, which indicates that the TPRs of the two racial groups are nearly equivalent. Bold value represents the best EEC-GIFT*-based LCS eligibility mechanism at the testing phase based on no significantly reduced predictive performance, the lowest average EOD, and the smallest effect size of the EOD.

## Discussion

Although accuracy and fairness are both key objectives when developing a fairness-aware ML framework to determine LCS eligibility, accuracy plays a more essential role than fairness, given that the values of TPR/FNR to measure fairness also depend on lung cancer predictive performance. Our bias mitigation strategy, GIFT, centered around equal opportunity or TPR/FNR. In the pre-processing process, we used a reweighing approach to evaluate the group weight coefficients based on sensitive attributes by pushing the value of SPD to 0. In the post-processing strategy, in addition to the threshold of “$T_{Y}$” generated by using equal weights to maximize the TPR and TNR of a ROC curve, we also used “$T_{U}$” to increase the number of individuals who qualify for LCS by adding more weight to the TPR than the TNR. The fairness notions in the GIFT strategy are compatible in terms of equal opportunity. Notably, though in-processing methods are integrated within the ML models themselves, pre- and post-processing methods can be applied across various classification methods. Thus, our fairness GIFT strategy is a model-agnostic approach and is compatible with a wide range of ML models regardless of their specific architecture or design.

Because most participants in our training dataset did not develop lung cancer, an ML model built on this data tends to be biased towards these smokers. Given the limitation of the LR model in handling class imbalance, we used the EEC model for lung cancer prediction. This model integrates 2 key components of random under-sampling and AdaBoost. The random under-sampling intends to address class imbalances, whereas AdaBoost focuses on instances with higher sample weights at each successive iteration and adjusts the weights of misclassified classes during each training iteration. Our numerical experiments showed that, with the same racial bias mitigation GIFT* strategy, the trained EEC-GIFT* model that considers the class imbalance issue significantly increased the fairness of LCS eligibility between Black and White smokers without compromising its accuracy. Thus, we consider the EEC-GIFT approach, which integrates the GIFT strategy into the EEC model, to be a systematic fairness-aware ML framework that can identify accurate and racially unbiased risk-based eligibility models from real-world data.

Our systematic fairness-aware ML framework is still in its infancy and has several limitations. First, one of our assumptions is that the base rates for sensitive lung cancer groups/subgroups are the same; thus, our approach can only be used when there is no significant base distribution difference among different sensitive groups/subgroups. Given that recent research has revealed significant racial disparities in lung cancer incidence rates between Black and White smokers,^36^ we plan to develop a more robust fairness-aware ML framework for LCS eligibility determination by employing error rate disparity to better define the fairness. Second, selection bias and missing data in real-world datasets can affect lung cancer predictions. Future research should account for these “true” imbalances and mitigate their impacts on the accuracy and fairness of the LCS eligibility models. Third, our study relied on a single dataset, which may not capture the variability encountered in broader clinical practice. The homogeneity of our data raises concerns about the models’ generalizability to other population samples, potentially limiting their applicability. Further validation with additional independent datasets is essential to ensure that the EEC-GIFT framework is robust and unbiased.

This study demonstrated the concept and development of our systematic fairness-aware ML framework. The framework could be used to develop an optimal LCS eligibility mechanism by considering more sensitive attributes, evaluating other advanced ML models that can handle class imbalances, and including additional eligibility threshold choices. Our framework can also be used for other types of cancer to identify appropriate screening eligibility mechanisms.

## Conclusion

This study used an EEC model, including random under-sampling and AdaBoost, to handle class imbalance issues in our training dataset and to predict lung cancer incidence for LCS eligibility identification. To mitigate racial biases in lung cancer incidence rates, we developed a fairness-enhancing GIFT strategy by integrating a pre-processing reweighing approach based on group or intersectional fairness and a post-processing eligibility threshold method with equal opportunity for Black and White participants. Applying the GIFT strategy to the EEC model resulted in a systematic fairness-aware ML framework, titled EEC-GIFT, for LCS eligibility determination. We identified LR-GIFT* and EEC-GIFT* as the best models for determining LCS eligibility in the training phase. Our testing results show that only the EEC-GIFT*–based LCS eligibility mechanism could significantly remove racial bias in the screening eligibility between Black and White participants without compromising the accuracy of lung cancer prediction. However, the systematic fairness-aware ML-based LCS eligibility model still needs to be validated with external independent datasets.

## Supplementary Material

pkaf030_Supplementary_Data

## Data Availability

The Prostate, Lung, Colorectal and Ovarian (PLCO) Cancer Screening Trial data can be accessed by submitting a project proposal to the Cancer Data Access System of National Cancer Institute (NCI) via https://cdas.cancer.gov/learn/plco/instructions/?type=data. Per the NCI’s Data Transfer Agreement, we are not allowed to share any data directly.

## References

[1] Siegel RL , Giaquinto AN, Jemal A. Cancer statistics, 2024. CA Cancer J Clin. 2024;74:12-49. 10.3322/caac.21820 38230766

[2] Aberle DR , Adams AM, Berg CD, et al Reduced lung-cancer mortality with low-dose computed tomographic screening. N Engl J Med. 2011;365:395-409. 10.1056/NEJMoa1102873 21714641 PMC4356534

[3] Moyer VA , US Preventive Services Task Force. Screening for lung cancer: U.S. Preventive Services Task Force recommendation statement. Ann Intern Med. 2014;160:330-338. 10.7326/M13-2771 24378917

[4] Tindle HA , Stevenson Duncan M, Greevy RA, et al Lifetime smoking history and risk of lung cancer: results from the Framingham Heart Study. J Natl Cancer Inst. 2018;110:1201-1207. 10.1093/jnci/djy041 29788259 PMC6235683

[5] Haddad DN , Sandler KL, Henderson LM, Rivera MP, Aldrich MC. Disparities in lung cancer screening: a review. Ann Am Thorac Soc. 2020;17:399-405. 10.1513/AnnalsATS.201907-556CME 32017612 PMC7175982

[6] Haiman CA , Stram DO, Wilkens LR, et al Ethnic and racial differences in the smoking-related risk of lung cancer. N Engl J Med. 2006;354:333-342. 10.1056/NEJMoa033250 16436765

[7] Robbins HA , Engels EA, Pfeiffer RM, Shiels MS. Age at cancer diagnosis for Blacks compared with Whites in the United States. J Natl Cancer Inst. 2015;107:dju489. 10.1093/jnci/dju489 PMC4326308 25638255

[8] Toumazis I , Bastani M, Han SS, Plevritis SK. Risk-based lung cancer screening: a systematic review. Lung Cancer. 2020;147:154-186. 10.1016/j.lungcan.2020.07.007 32721652

[9] Krist AH , Davidson KW, Mangione CM, et al Screening for lung cancer: US Preventive Services Task Force Recommendation Statement. JAMA. 2021;325:962-970. 10.1001/jama.2021.1117 33687470

[10] Aredo JV , Choi E, Ding VY, et al Racial and ethnic disparities in lung cancer screening by the 2021 USPSTF guidelines versus risk-based criteria: the multiethnic cohort study. JNCI Cancer Spectr. 2022;6:pkac033. 10.1093/jncics/pkac033 PMC9156850 35642317

[11] Pinheiro LC , Groner L, Soroka O, et al Analysis of eligibility for lung cancer screening by race after 2021 changes to US Preventive Services Task Force Screening Guidelines. JAMA Netw Open. 2022;5:e2229741. 10.1001/jamanetworkopen.2022.29741 36053535 PMC9440399

[12] Tammemagi MC , Katki HA, Hocking WG, et al Selection criteria for lung-cancer screening. N Engl J Med. 2013;368:728-736. 10.1056/NEJMoa1211776 23425165 PMC3929969

[13] Mitchell S , Potash E, Barocas S, D'Amour A, Lum K. Algorithmic fairness: choices, assumptions, and definitions. Annu Rev Stat Its Appl. 2021;8:141-163.

[14] Chouldechova A , Roth A. A snapshot of the frontiers of fairness in machine learning. *Commun ACM*. 2020;63:82-89.

[15] Friedler SA , Scheidegger CE, Venkatasubramanian S, Choudhary S, Hamilton EP, Roth D. A comparative study of fairness-enhancing interventions in machine learning. *Proceedings of the Conference on Fairness, Accountability, and Transparency (FAT* '19)*. 2019:329-338.

[16] Choi E , Ding VY, Luo SJ, et al Risk model-based lung cancer screening and racial and ethnic disparities in the US. JAMA Oncol. 2023;9:1640-1648. 10.1001/jamaoncol.2023.4447 37883107 PMC10603577

[17] Pessach D , Shmueli E. Algorithmic fairness. Machine Learning for Data Science Handbook: Data Mining and Knowledge Discovery Handbook. Springer International Publishing. 2023:867-886.

[18] Ovalle A , Subramonian A, Gautam V, Gee G, Chang K-W. Factoring the matrix of domination: a critical review and reimagination of intersectionality in AI fairness. *AAAI/ACM Conference on AI, Ethics, and Society (AIES '23)*. August 08-10, 2023:496-511.

[19] Kearns M , Neel S, Roth A, Wu ZS. Preventing fairness gerrymandering: auditing and learning for subgroup fairness. *Proceedings of the 35th International Conference on Machine Learning*, Vol. 80. PMLR; 2018:2564-2572.

[20] Mahoney T , Varshney KR, Hind M. AI Fairness: How to Measure and Reduce Unwanted Bias in Machine Learning. O'Reilly Media, Inc.; 2020.

[21] Kamiran F , Calders T. Data preprocessing techniques for classification without discrimination. Knowl Inf Syst. 2012;33:1-33. 10.1007/s10115-011-0463-8

[22] Ahang B , Lemoine B, Mitchell M. Mitigating unwanted biases with adversarial learning. *Proceedings of the 2018 AAAI/ACM Conference on AI, Ethics, and Society (AIES '18)*. 2018:335-340.

[23] Hardt M , Price E, Srebro N. Equality of opportunity in supervised learning. *Proceedings of the 30th International Conference on Neural Information Processing Systems (NIPS '16)*. 2016:3323-3331.

[24] Wesson P , Hswen Y, Valdes G, Stojanovski K, Handley MA. Risks and opportunities to ensure equity in the application of big data research in public health. Annu Rev Publ Health. 2022;43:59-78. 10.1146/annurev-publhealth-051920-110928 PMC8983486 34871504

[25] Prorok PC , Andriole GL, Bresalier RS, et al Design of the Prostate, Lung, Colorectal and Ovarian (PLCO) cancer screening trial. Control Clin Trials. 2000;21:273S-309S. 10.1016/s0197-2456(00)00098-2 11189684

[26] Tanha J , Abdi Y, Samadi N, Razzaghi N, Asadpour M. Boosting methods for multi-class imbalanced data classification: an experimental review. J Big Data. 2020;7:1–47. ARTN 70 10.1186/s40537-020-00349-y

[27] Liu XY , Wu J, Zhou ZH. Exploratory undersampling for class-imbalance learning. IEEE Trans Syst Man Cybern B Cybern. 2009;39:539-550. 10.1109/TSMCB.2008.2007853 19095540

[28] Youden WJ. Index for rating diagnostic tests. Cancer. 1950;3:32-35. 10.1002/1097-0142(1950)3:1<32::aid-cncr2820030106>3.0.co;2-3 15405679

[29] McNemar Q. Note on the sampling error of the difference between correlated proportions or percentages. Psychometrika. 1947;12:153-157. 10.1007/Bf02295996 20254758

[30] DeLong ER , DeLong DM, Clarke-Pearson DL. Comparing the areas under 2 or more correlated receiver operating characteristic curves—a nonparametric approach. Biometrics. 1988;44:837-845. 10.2307/2531595 3203132

[31] Lakens D. Calculating and reporting effect sizes to facilitate cumulative science: a practical primer for t-tests and ANOVAs. Front Psychol. 2013;4:863. 10.3389/fpsyg.2013.00863 24324449 PMC3840331

[32] McGraw KO , Wong SP. A common language effect size statistic. Psychol Bull. 1992;111:361-365. 10.1037/0033-2909.111.2.361

[33] Parkin DM , Moss SM. Lung cancer screening—improved survival but no reduction in deaths—The role of "overdiagnosis". Cancer. 2000;89:2369-2376. 10.1002/1097-0142(20001201)89:11+<2369::aid-cncr10>3.0.co;2-a 11147614

[34] Marcus PM , Bergstralh EJ, Zweig MH, Harris A, Offord KP, Fontana RS. Extended lung cancer incidence follow-up in the Mayo Lung Project and overdiagnosis. J Natl Cancer I. 2006;98:748-756. 10.1093/jnci/djj207 16757699

[35] Katki HA , Kovalchik SA, Berg CD, Cheung LC, Chaturvedi AK. Development and validation of risk models to select ever-smokers for CT lung cancer screening. JAMA-J Am Med Assoc. 2016;315:2300-2311. 10.1001/jama.2016.6255 PMC4899131 27179989

[36] Stram DO , Park SL, Haiman CA, et al Racial/ethnic differences in lung cancer incidence in the multiethnic cohort study: an update. J Natl Cancer Inst. 2019;111:811-819. 10.1093/jnci/djy206 30698722 PMC6695313

