# Supplementary material for: Easy ensemble classifier-group and intersectional fairness and threshold (EEC-GIFT): a fairness-aware machine learning framework for lung cancer screening eligibility using real-world data
Source: JNCI Cancer Spectr. 2025 Mar 20;9(2):pkaf030. doi: 10.1093/jncics/pkaf030 (PMC11986816; doi:10.1093/jncics/pkaf030)

**Supplementary Materials**

**Supplementary Methods**

**Equal Opportunity Difference for Fairness Measurement**

Though satisfying both true positive (TP) parity and false positive (FP) parity constraints can make the equalized odds as fair as possible, fairness interventions intend to equalize outcomes across different groups, which may increase fairness and significantly decrease accuracy for individual outcome prediction.^1^ This indicates that there is an inherent trade-off between accuracy and fairness for any ML-based outcome prediction model, including an LCS eligibility model. Given that the sum of TP rate (TPR) and false negative rate (FNR) is one in terms of incidence prediction, their values are known once one of them is evaluated. The pair of FP rate (FPR)/true negative rate (TNR) has the same property. As a relaxed version of equalized odds, equal opportunity to evaluate the fairness of Black and White smokers’ LCS eligibility focuses only on TPRs/FNRs, an LCS eligibility mechanism developed based on equal opportunity only slightly compromises the fairness of the LCS eligibility model between two racial groups generated from the equalized odds. As a tradeoff, it can improve the accuracy of lung cancer prediction model because there is no FPR/TNR constraint on it. Importantly, equal opportunity is a reasonable way to measure group fairness in many real-world clinical practices. In LCS, failure to alert a smoker who is at risk of lung cancer can result in a FN diagnosis, leading to a higher disease stage-at-diagnosis and an increased risk of death; however, the impact of FP diagnoses became trivial with the invention of the LDCT. As such, in clinical practice, measuring TPR/FNR is much more important than measuring FPR/TNR, and replacing equalized odds with equal opportunity allows for higher prediction accuracy without significantly compromising fairness. Thus, we used equal opportunity to define fairness across Black and White participant groups in this study and used average equal opportunity difference (EOD) to measure and compare the fairness of LCS eligibility mechanisms.

**Data Preparation and Feature Selection**

Data for this study came from the PLCO dataset, a randomized controlled trial that intended to determine whether certain screening exams reduce mortality from prostate, lung, colorectal, and ovarian cancers; participants were randomly assigned to either the control or intervention arm in equal proportions. The median follow-up time of cancer diagnoses were 11.3 years, and the median follow-up for mortality was 19.2 years. Out of 251 total variables, we used expert knowledge to curate 30 features that were relevant to lung cancer incidence. These selected features spanned across four categories: (1) *demographic factors*, including “age at trial entry”, “gender”, “race”, “education level”, “marital status”, “occupation status”, and “BMI at baseline”; (2) *smoking history*, including “cigarette smoking status”, “number of years since the participant stopped smoking cigarettes”, “how long the participant smoked cigarettes”, “number of cigarettes smoked per day”, “pack-years”, and “history of cigar or pipe smoking”; (3) *medical history and* *lung-related issues*, including “chronic bronchitis”, “emphysema”, “family history of lung cancer”, “personal history of any cancer”, and “chest X-ray history”; and (4) *other medical comorbidities*, including “arthritis”, “colon-related co-morbidities”, “diabetes”, “diverticulosis or diverticulitis”, “gallstones or gallbladder inflammation”, “coronary heart disease or a heart attack”, “hypertension”, “liver-related co-morbidity (e.g., hepatitis or cirrhosis)”, “osteoporosis”, “colorectal polyps”, and “stroke”. Additionally, to facilitate feature selection, we aggregated a new ordinal feature, “COPD”, by merging the binary comorbidities of “chronic bronchitis” and “emphysema”. We assigned a value of 2 if both conditions were present, 1 if either condition was present, and 0 if neither condition was present. Similarly, we also generated the new ordinal feature of “Personal Health History” by aggregating the two binary historical features of “family history of lung cancer”, “personal history of any cancer”, and “chest X-ray history” given their potential relationship with lung cancer. Then, the values of this new feature were assigned between 0 to 3 based on the number of historical features found in a participant’s records.

We performed a feature selection process to identify the optimal set of informative features by using univariate statistics in conjunction with the sequential forward selection. The chi-squared test was used to determine the association between each categorical feature and lung cancer incidence, and the ANOVA F-values were used to examine the significance of differences among means of numerical features across target classes for univariate statistical tests. The features with *P*<0.05 were retained and then used in a sequential forward selection method to select a final set of important features for the lung cancer prediction model. The sequential forward feature selection process started with an empty set of features and iteratively added one feature at a time, selecting the features that most enhances lung cancer incidence prediction. The selection process ceased when model performance declined. Given the potential of pre-processing to alter the original dataset, we conducted feature selection before the pre-processing process to develop the EEC-based LCS eligibility mechanism.

**Pre-Processing Reweighing Approach**

This study used the SPD metric to detect bias and a reweighing approach to mitigate the bias by calculating and assigning different weights to instances in the training/testing dataset. Of the many potential methods for the pre-processing strategy, we selected the reweighing technique for its simplicity and non-intrusive nature. It additionally preserved the original data labels for race and lung cancer incidence, ensuring that the input data remained as impartial as possible. We evaluated the reweighing approach for both group fairness (one sensitive attribute: race) and intersectional fairness (two sensitive attributes: race and gender) and selected the one that achieved the best accuracy and fairness results.

Let *Y* represents the diagnosis outcome of whether a participant has lung cancer or not, in which the class “Positive” or “Negative” indicate diagnosis outcome yes or no. The sensitive attribute of race only focuses on “Black” and “White” racial groups. Let class probability $\Pr\left( Y=Positive|race=Black \right)$ or $\Pr(Y=Positive|race=White)$ represent the probability of Black or White participants developing lung cancer (class = “Positive”). The value of the *SPD* between two groups can be computed from the following equation:

${SPD}_{Positive|Black, White}=\Pr\left( Y=Positive|race=Black \right)-\Pr(Y=Positive|race=White)$ (1)

For the group fairness, our reweighing method evaluates weights for four race-incidence subgroups generated from the combination of the sensitive attribute (“Black” and “White”) and class label (“Positive” or “Negative”) to remove the racial disparities in the dataset. These weights were treated as the frequency of re-distributing smokers in original race-incidence groups to generate a training dataset with the same incidence rates of Blacks and Whites for LCS eligibility mechanism determination.

Let $N_{Black, Positive}$ and $N_{Black, Negative}$ or $N_{White, Positive}$, and $N_{White, Negative}$ indicate the number of Black or White participants with and without lung cancer in the training dataset, and $N_{Black}$ or $N_{White}$ indicate the number of Black or White participants. As such, $N_{Black}=N_{Black, Positive}+N_{Black, Negative}$ or $N_{White}=N_{White, Positive}+N_{White, Negative}$. $N_{Positvie}$ or $N_{Negative}$ indicated the number of smokers with lung cancer diagnosis (yes or no), resulting in $N_{Positive}=N_{Black, Positive}+N_{White,Positive}$ or $N_{Negative}=N_{Black,Negative}+N_{White, Negative}$. *N* indicated the total number of smokers in the training/testing dataset, resulting in ${N=N}_{Positive}+N_{Negative}=$ $N_{Black}+N_{White}$. Assuming that race and lung cancer diagnosis are independent, the weight coefficient $W_{Black, Positive}$ of Black smokers with lung cancer can be calculated from the ratio of the expected probability of this group to its corresponding observed probability ^2^ from the following equation:

$W_{Black, Positive}= \frac{N_{Black} * N_{Positive}}{N *N_{Black, Positive}}$, (2)

where the other weights of $W_{Black, Negative}$, $W_{White, Positive}$, and $W_{White, Negative}$ can also be calculated similarly. After using these weights to update the frequencies of each group in our training/testing dataset, the SPDs computed from the updated dataset would approach to 0, indicating that the lung cancer diagnosis rate is free of racial discrimination.

The above strategy of bias detection and reweighing coefficient evaluation for group fairness can be extended to intersectional fairness by considering two or more sensitive attributes. Though the number of possible subgroups depends on these sensitive variables, the biases in lung cancer incidence between any two subgroups can be detected from their corresponding SPDs, and reweighing coefficients for each subgroup (with and without lung cancer) can also be evaluated from the ratio of the expected probability of this subgroup to its corresponding observed probability based on our training/testing dataset. For example, we considered the two sensitive attributes of race (“Black” and “White”) and gender (“Male” and “Female”) with four resulting subgroups of “White Male”, “White Female”, “Black Male”, and “Black Female”. The equations to detect the bias between “Black Female” and “White Female” subgroups and to evaluate the reweighing coefficients of the “Black Female” subgroup with lung cancer can be described as follows.

Let $\Pr\left( Y=Positive|race=Black, gender=Female \right)$ and $\Pr(Y=Positve|race=White, gender=Female)$ indicate the class probabilities of the “Black Female” and “White Female” subgroups developing lung cancer, respectively. The value of the *SPD* to detect the disparities in lung cancer incidence rate between them can be computed from the following equation:

${SPD}_{Positive|Black\_Female, White\_Female}=\Pr\left( Y=Positve|race=Black,gender=Female \right)-\Pr(Y=Positive|race=White, gender=Female)$, (3)

where the value of the SPD between the other two subgroups (“Black Male” and “White Male”) can be calculated similarly. Let $N_{Black,Female, Positive}$ indicate the number of smokers diagnosed with lung cancer in the “Black Female” subgroup and $N_{Black,Female}$ indicate the number of participants in this subgroup. Assuming that race, gender, and lung cancer diagnosis are independent, the weight coefficient $W_{Black, Female, Positive}$ of smokers with lung cancer in the subgroup can be calculated from the following equation:

$W_{Black, Female,Positive}= \frac{N_{Black,Female} * N_{Positive}}{N *N_{Black, Female,Positive}}$, (4)

where the weight coefficients of other subgroups with or without lung cancer can be computed similarly. After using these weight coefficients to update the frequencies of each subgroup in our training/testing dataset, the SPDs calculated from the updated dataset would approach to 0, indicating that the lung cancer diagnosis rate is free of discrimination for that subgroup.

**Easy Ensemble Classifier (EEC)-based Lung Cancer Predictive Model**

The EEC consisted of two iterative steps: ^3^ 1) random under-sampling was used to handle the imbalanced lung cancer incidence rate and the ratio of Black to White smokers to generate diverse balanced bootstrap samples; 2) the AdaBoost classifier ^4^ was used as the base estimator trained on multiple sets of balanced bootstrap samples by generating multiple weak learners from the bootstrap samples and combining them to create a strong classifier. AdaBoost learners work by putting more weight on difficult-to-classify instances and less weight on instances that are already handled well. The final prediction is made by calculating the weighted sum of the predictions from all the weak learners. We used a grid search to tune the hyperparameters of the EEC-based prediction model by using stratified 10-fold cross-validation (CV) to identify the EEC-based lung cancer prediction model that display the most accurate lung cancer prediction.

Though the fair EEC model associated with the pre-processing bias mitigation strategy also consists of iterative random under-sampling and the AdaBoost classifier, its AdaBoost algorithm is different from that of the EEC model. Our pre-processing strategy incorporates the weight coefficients evaluated from equations similar to Equation (2) or (4) into training samples within each group or subgroup at the beginning of the process. These initial weights set the algorithm’s initial focus, influencing which instances are considered more important to classify correctly from the outset. In each round, a weak learner is trained on the data, and its error is calculated based on the weights of the training instances. AdaBoost adjusts the weights of the instances, but these adjustments are based on the initial weights provided. For misclassified instances, their weights are increased according to the formula that factors in the total weights of the misclassified instances of the current learner,^4^ ensuring that the increase is proportional to their original weights. Conversely, the weights of correctly classified instances are adjusted downward, also relative to their starting weights. These steps are repeated for a specified number of estimators, and the final model is a weighted sum of the weak learners.

**References**

1. Buijsman S. Navigating Fairness Measures and Trade-Offs. AI and Ethics Springer; 2023.
2. Kamiran F, Calders T. Data preprocessing techniques for classification without discrimination. Knowledge and Information Systems. 2012;33(1):1-33.
3. Liu XY, Wu J, Zhou ZH. Exploratory undersampling for class-imbalance learning. IEEE Trans Syst Man Cybern B Cybern. 2009;39(2):539-50.
4. Schapire RE. A Brief Introduction to Boosting. International Joint Conference on Artificial Intelligence1999.
5. Youden WJ. Index for rating diagnostic tests. Cancer. 1950;3(1):32-5.
6. USPSTF, Krist AH, Davidson KW, Mangione CM, Barry MJ, Cabana M, et al. Screening for Lung Cancer: US Preventive Services Task Force Recommendation Statement. JAMA. 2021;325(10):962-70.
7. Delong ER, Delong DM, Clarkepearson DI. Comparing the Areas under 2 or More Correlated Receiver Operating Characteristic Curves - a Nonparametric Approach. Biometrics. 1988;44(3):837-45.
8. McNemar Q. Note on the Sampling Error of the Difference between Correlated Proportions or Percentages. Psychometrika. 1947;12(2):153-7.

**Supplementary Table 1. The glossary of technical terms and their definitions.**

| **Term** | **Definition** |
| --- | --- |
| Area Under the ROC Curve (AUC) | A metric for evaluating the performance of classification models across different threshold settings. The ROC is a probability curve, and the AUC represents the degree of separability. A higher AUC score indicates a greater capability of the model to correctly distinguish between positive and negative classes, with the model accurately predicting 0s as 0s and 1s as 1s. |
| Bootstrapping | A statistical technique that involves resampling with replacement from a dataset to create multiple new datasets (bootstrap samples). These samples are used to estimate the distribution of a statistic or to train multiple instances of a ML model in ensemble methods. Bootstrapping helps in assessing the variability of the model and improving its robustness. |
| Classifier | An ML model or algorithm is used to categorize data into different classes, for example, 0-Not eligible and 1-Eligible. |
| Confidence Interval (CI) | The mean of an estimate +/- the variation in the estimate. |
| DeLong Test | A statistical method used to evaluate the difference in the AUC score between the two models. The test accounts for the correlation between the ROC curves by computing the variance of the AUC differences and determining if one model significantly outperforms the other. |
| Effect Size | A statistical measure that represents the magnitude of the difference between two groups on a particular variable, expressed in terms of standard deviations. |
| Ensemble Learning | Combining the predictions of multiple models to achieve a more accurate result than any individual model. The three main methods include bagging, boosting, and stacking. A boosting technique, such as AdaBoost, involves adding ensemble members sequentially that correct the predictions made by prior models and output a weighted average of the predictions. |
| F1-score | A measure of a model's accuracy that considers both precision and recall. It is the harmonic mean of precision and recall. The F1-score ranges from 0 to 1, with 1 being the best possible score. |
| Fairness | Ensuring that a ML model’s predictions are unbiased and equitable across different groups defined by sensitive attributes like race and/or gender. |
| Fairness Gerrymandering | The phenomenon where a ML model appears to be fair overall but discriminates against certain subgroups within the data. This occurs when fairness metrics are optimized for the entire population but certain intersections of attributes (e.g., gender and race) experience significant bias. |
| False Negative Rate (FNR) | The proportion of actual positives that are incorrectly identified as negatives by the model. It is calculated as the number of false negatives (FN) divided by the total number of actual positives (TP + FN). |
| False Positive Rates (FPR) | The proportion of actual negatives that are incorrectly identified as positives by the model. It is calculated as the number of false positives (FP) divided by the total number of actual negatives (TN + FP). |
| Grid search Hyperparameter Tuning | The process of finding the optimal hyperparameters for a ML model. It involves systematically searching through a manually specified subset of the hyperparameter space of the learning algorithm. The performance of the model is evaluated for each combination of parameters, and the set that results in the best performance is selected. This process helps in improving the accuracy and efficiency of the model. |
| *K*-Fold Cross Validation (CV) | A technique used to evaluate the performance of a ML model. The data set is randomly divided into *K* (e.g., 10) equally sized subsets or "folds." The model is trained K times, each time using *K*-1 folds for training and the remaining fold for testing. The average performance across all *K* trials is used as the final evaluation metric. |
| Kolmogorov-Smirnov Normality Test | A nonparametric statistical test that quantifies a distance between the empirical distribution function of the sample with the distribution expected if the data were normal. |
| Machine Learning (ML) | A field of study in artificial intelligence concerned with the development and study of statistical algorithms that can learn from data and generalize to unseen data and thus perform tasks without explicit instructions. |
| McNemar Test | A statistical test used to compare the performance of two models in terms of their error rates, focusing on cases where the models make different predictions. |
| Pack-Year | A method to quantify an individual's long-term smoking exposure. It is calculated by multiplying the number of packs of cigarettes smoked per day by the number of years the person has smoked. For example, 1 pack-year corresponds to smoking one pack of cigarettes per day for one year, or two packs per day for half a year. |
| Random Under-Sampling | A technique used to address class imbalance in a dataset by randomly removing samples from the majority class to match the size of the minority class. |
| Risk-based Eligibility Model | A predictive model used to predict the likelihood of a patient developing a condition like lung cancer, and based on this risk, decide his/her eligibility for screening (1-Yes, 0-No) |
| Receiver Operating Characteristic Curve (ROC Curve) | A graphical plot that illustrates the diagnostic ability of a binary classifier as its discrimination threshold is varied. It plots the true positive rate against the false positive rate. |
| Stratified *K*-fold Cross Validation (CV) | A variation of *K*-Fold CV where each fold is created in such a way that the proportion of samples for each class is approximately the same as in the original dataset. |
| Threshold | A risk decision boundary that determines a smoker’s LCS eligibility (Yes/No) based his/her predicted lung cancer incidence probability. For instance, if the risk threshold is set at 0.2, all smokers with a predicted incidence probability equal to and above 0.2 are classified as eligible to LCS, and those below are classified as ineligible to LCS. |
| True Negative Rate (TNR) | The proportion of actual negatives that are correctly identified by the ML model. It is calculated as the number of true negatives (TN) divided by the total number of actual negatives (TN + FP). It is also known as Specificity. |
| True Positive Rate (TPR) | The proportion of actual positives that are correctly identified by the ML model. It is calculated as the number of true positives (TP) divided by the total number of actual positives (TP + FN). It is also known as Sensitivity or Recall. |
| Weak Learners | Models that perform slightly better than random guessing. In the context of ensemble methods, such as boosting, multiple weak learners are combined to form a strong learner. Each weak learner focuses on different parts of the data or errors made by previous learners, and their combined predictions improve the overall performance of the model. |

**Supplementary Table 2**. Weight coefficients ^a^ based on pre-processing strategy RW1 for the training and testing datasets.

| Sub-Group ^b^ | Race | Lung Cancer | Training Dataset | | | | Testing Dataset | | | |
| --- | --- | --- | --- | --- | --- | --- | --- | --- | --- | --- |
|  |  |  | Before Reweighing | | After Reweighing | | Before Reweighing | | After Reweighing | |
|  |  |  | Count | Class Probability | **Weight Coefficients** | Class Probability | Count | Class Probability | **Weight Coefficients** | Class Probability |
| RW1-1 | White | Positive | 1290 | 0.0410 | **1.0048** | 0.0412 | 1354 | 0.0420 | **1.0221** | 0.043 |
| RW1-2 | White | Negative | 30197 |  | **0.9998** |  | 30873 |  | **0.9990** |  |
| RW1-3 | Black | Positive | 85 | 0.0444 | **0.9279** | 0.0412 | 115 | 0.0580 | **0.7401** | 0.043 |
| RW1-4 | Black | Negative | 1831 |  | **1.0034** |  | 1867 |  | **1.0160** |  |
| SPD (Before Reweighing) ^c^ | | | 0.0444 - 0.0410 =0.0034 | | | | 0.058 - 0.042 = 0.016 | | | |
| SPD (After Reweighing) ^d^ | | | ((85*0.9279)/1916) - ((1290*1.0048)/31487) ~ 0 | | | | ((115*0.7401)/1982) - ((1354*1.0221)/32227) ~ 0 | | | |

^a^ The bold value represents the weight coefficients for the RW1 pre-processing strategy, and they are computed from Equations (2) for both the training and testing datasets.

^b^ Four subgroups were derived from the combination of Black/White smokers, with/without lung cancer incidence, under the RW1 reweighing approach with a single sensitive attribute (“race”).

^c^ The SPD values, computed from Equations (1) before applying the reweighing approach, reveal that racial biases between subgroups exist in both the training and testing datasets, with a particularly high bias observed in the testing dataset.

^d^ The SPD values, computed from Equation (1) after applying the reweighing approach, indicate that the updated training and testing datasets according to the weight coefficients are free of racial discrimination regarding positive lung cancer diagnosis.

**Supplementary Table 3**. Weight coefficients ^a^ based on pre-processing strategy RW2 for the training and testing datasets.

| Sub-Group ^b^ | Race | Gender | Lung Cancer | Training Dataset | | | | Testing Dataset | | | |
| --- | --- | --- | --- | --- | --- | --- | --- | --- | --- | --- | --- |
|  |  |  |  | Before Reweighing | | After Reweighing | | Before Reweighing | | After Reweighing | |
|  |  |  |  | Count | Class Probability (CP) | **Weight Coefficients** | Class Probability | Count | Class Probability (CP) | **Weight Coefficients** | Class Probability |
| RW2-1 | White | Male | Positive | 796 | 0.0436 | **0.9442** | 0.0412 | 840 | 0.0447 | **0.9596** | 0.0429 |
| RW2-2 | White | Male | Negative | 17463 |  | **1.0025** |  | 17932 |  | **1.0019** |  |
| RW2-3 | White | Female | Positive | 494 | 0.0373 | **1.1023** | 0.0412 | 514 | 0.0382 | **1.1241** | 0.0429 |
| RW2-4 | White | Female | Negative | 12734 |  | **0.9960** |  | 12941 |  | **0.9951** |  |
| RW2-5 | Black | Male | Positive | 45 | 0.0455 | **0.9047** | 0.0412 | 83 | 0.0774 | **0.5551** | 0.0429 |
| RW2-6 | Black | Male | Negative | 944 |  | **1.0045** |  | 990 |  | **1.0373** |  |
| RW2-7 | Black | Female | Positive | 40 | 0.0431 | **0.9540** | 0.0412 | 32 | 0.0352 | **1.2198** | 0.0429 |
| RW2-8 | Black | Female | Negative | 887 |  | **1.0021** |  | 877 |  | **0.9920** |  |
| SPD (Before Reweighing) ^c^ | | | | \|CP (White Male) - CP (White Female) \| = 0.0063  \|CP (White Male) - CP (Black Male) \| = 0.0019  \|CP (White Male) - CP (Black Female) \| = 0.0005  \|CP (White Female) - CP (Black Male) \| **= 0.0082**  \|CP (White Female)- CP (Black Female) \| = 0.0058  \|CP (Black Male) - CP (Black Female) \| = 0.0024 | | | | \|CP (White Male) - CP (White Female) \| = 0.0065  \|CP (White Male) - CP (Black Male) \| = 0.0326  \|CP (White Male) - CP (Black Female) \| = 0.0095  \|CP (White Female) - CP (Black Male) \| **= 0.0392**  \|CP (White Female) - CP (Black Female) \| = 0.003  \|CP (Black Male) - CP (Black Female) \| = 0.0422 | | | |
| SPD (After Reweighing) ^d^ | | | | White Male: (796*0.9442)/18259 = 0.0412  White Female: (494*1.1023)/13228 =0.0412  Black Male: (45*0.9047)/989= 0.0412  Black Female: (40*0.954)/927 =0.0412  SPD (every two subgroups) ~ 0 | | | | White Male: (840*0.9596)/18772= 0.0429  White Female: (514*1.1241)/13455=0.0429  Black Male: (83*0.5551)/1073= 0.0429  Black Female: (32*1.2198)/909 =0.0429  SPD (every two subgroups) ~ 0 | | | |

^a^ The bold value represents the weight coefficients for the RW2 pre-processing strategy, and they are computed from Equation (4) for both the training and testing datasets.

^b^ Eight subgroups were created from the combination of male/female, Black/White smokers, with/without lung cancer incidence, under the RW2 reweighing approach, with two sensitive attributes (race and gender),

^c^ The SPD values were computed from Equation (3) before applying the reweighing approach. The pairwise comparison of four groups, “White Male”, “White Female”, “Black Male”, and “Black Female” resulted in six differences in total, and the highest value among these differences (indicated in bold) is reported as the SPD value. This value reveals that racial biases exist in both the training and testing datasets, with a particularly high bias observed in the testing dataset.

^d^ The SPD values computed from Equation (3) after applying the reweighing approach, indicate that the updated training and testing datasets according to the weight coefficients are free of racial discrimination regarding positive lung cancer diagnosis.

# Supplementary Figure 1: Threshold identification in our post-processing strategy.

In the EEC-GIFT-based LCS eligibility model, the risk of a smoker developing lung cancer can be estimated after applying the pre-processing reweighing approach to the EEC-based lung cancer prediction model. By comparing the risk with the true value, the prediction performance of the EEC-GIFT model can be evaluated from the value of the area under the receiver operating characteristic (ROC) curve (AUC). The AUC is one of the most important evaluation metrics to check the performance of classification models, in which an AUC of 0.5 represents random guessing and an AUC of 1 indicates perfect prediction. However, evaluating the model’s fairness requires a lung cancer risk threshold to determine whether a smoker is eligible for LCS, which can be determined by comparing the probability of their developing lung cancer to the threshold. Our post-processing strategy intends to identify the best threshold to determine the EEC-GIFT-based LCS eligibility mechanism with the highest fairness.

Given that equal opportunity/TPR was used in this study to measure fairness, we defined a range of potential thresholds from 0 to “$T_{Y}$”, which is a threshold associated with the Youden Index^5^ of an ROC curve to optimally balance the trade-off between TPR and TNR. We were especially interested in testing the fairness of the EEC-GIFT-based LCS eligibility model via the threshold “$T_{U}$”, which is a threshold associated with the 2021 US Preventive Services Task Force (USPSTF) criteria.^6^ Using “$T_{U}$” will allow us to keep the model’s specificity the same as that of the 2021 USPSTF criteria. The values of thresholds “$T_{Y}$” and “$T_{U}$” can be identified from the ROC curve of EEC-GIFT-based lung cancer prediction model during the training phase as shown in this figure. After evaluating the sensitivity and specificity of the 2021 USPSTF criteria and locating the point of “Specificity of USPSTF Criteria” based on them, the value of threshold “$T_{U}$” can be identified from the intersection of the ROC curve and the extended vertical line above the node “Specificity of 2021 USPSTF Criteria”. As a common summary measure of the ROC curve, the Youden Index indicates the maximum potential effectiveness of the EEC-GIFT-based lung cancer prediction model. We then evaluated the value of threshold “$T_{Y}$” from the cut-point of the ROC curve in this figure that optimized the prediction model’s differentiating ability, with equal weight given to sensitivity and specificity.


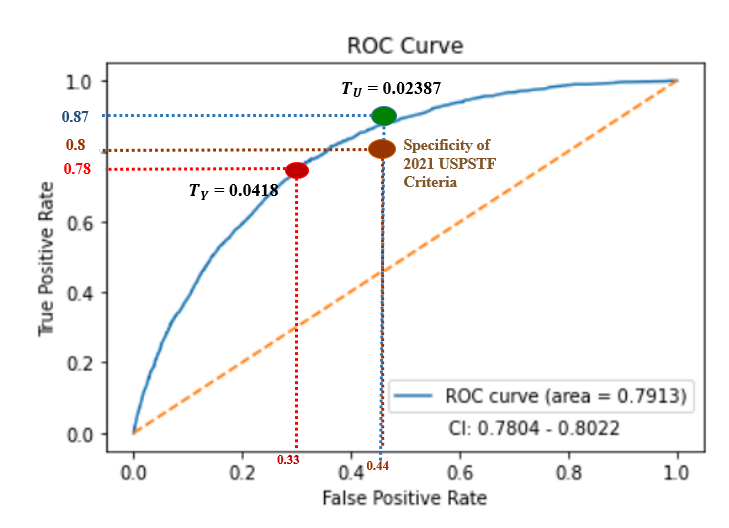


# Supplementary Figure 2: The importance of features to determine the risk of lung cancer based on the EEC-GIFT* model.

The best hyperparameters of the EEC-GIFT* model consisted of 70 different AdaBoost learners, 200 weak learners for each AdaBoost ensemble, and a learning rate of 0.1. After evaluating each feature’s average contribution across all the AdaBoost classifiers in the ensemble, we considered the average contribution as each feature’s importance score to quantify its contribution in minimizing uncertainty or impurity from the trained EEC-GIFT*-based LCS eligibility model. Our numerical experiments show that “pack-years”, “current BMI”, “the number of years that the participant has smoked”, “participant age”, and “the number of years since the participant stopped smoking” are the five most influential features to determine lung cancer risk.


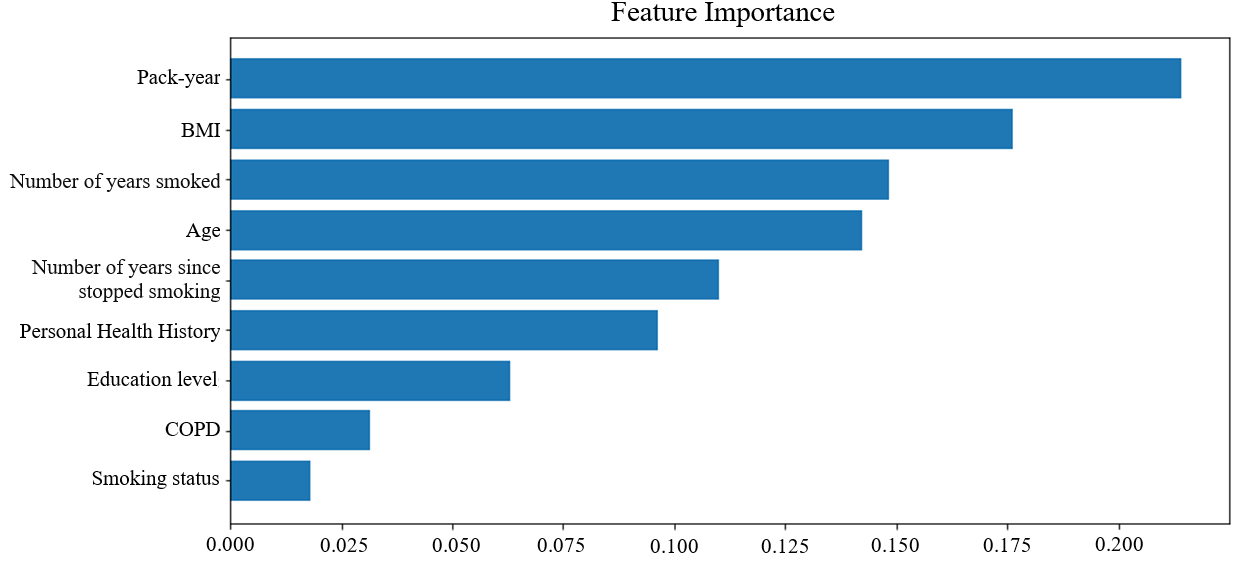

Supplement: pkaf030_Supplementary_Data [file pkaf030_supplementary_data.zip › Supplementary Material_JNCI_R1_final.docx]
